# Catalytic patch with redox Cr/CeO_2_ nanozyme of noninvasive intervention for brain trauma

**DOI:** 10.7150/thno.51912

**Published:** 2021-01-01

**Authors:** Shaofang Zhang, Ying Liu, Si Sun, Junying Wang, Qifeng Li, Ruijuan Yan, Yalong Gao, Haile Liu, Shuangjie Liu, Wenting Hao, Haitao Dai, Changlong Liu, Yuanming Sun, Wei Long, Xiaoyu Mu, Xiao-Dong Zhang

**Affiliations:** 1 Tianjin Key Laboratory of Low Dimensional Materials Physics and Preparing Technology, Institute of Advanced Materials Physics, School of Sciences, Tianjin University, Tianjin, 300350, China.; 2 Academy of Medical Engineering and Translational Medicine, Medical College, Tianjin University, Tianjin, 300072, China.; 3 Tianjin Key Laboratory of Molecular Nuclear Medicine, Institute of Radiation Medicine, Chinese Academy of Medical Sciences and Peking Union Medical College, Tianjin, 300192, China.; 4 Department of Neurosurgery and Key Laboratory of Post-trauma Neuro-repair and Regeneration in Central Nervous System, Tianjin Medical University General Hospital, Tianjin 300052, China.

**Keywords:** nanozyme, multienzyme-like activity, RONS-scavenging, traumatic brain injury, noninvasive treatment

## Abstract

Traumatic brain injury (TBI) is a sudden injury to the brain, accompanied by the production of large amounts of reactive oxygen and nitrogen species (RONS) and acute neuroinflammation responses. Although traditional pharmacotherapy can effectively decrease the immune response of neuron cells via scavenging free radicals, it always involves in short reaction time as well as rigorous clinical trial. Therefore, a noninvasive topical treatment method that effectively eliminates free radicals still needs further investigation.

**Methods:** In this study, a type of catalytic patch based on nanozymes with the excellent multienzyme-like activity is designed for noninvasive treatment of TBI. The enzyme-like activity, free radical scavenging ability and therapeutic efficacy of the designed catalytic patch were assessed *in vitro* and *in vivo*. The structural composition was characterized by the X-ray diffraction, X-ray photoelectron spectroscopy and high-resolution transmission electron microscopy technology.

**Results:** Herein, the prepared Cr-doped CeO_2_ (Cr/CeO_2_) nanozyme increases the reduced Ce^3+^ states, resulting in its enzyme-like activity 3-5 times higher than undoped CeO_2_. Furthermore, Cr/CeO_2_ nanozyme can improve the survival rate of LPS induced neuron cells via decreasing excessive RONS. The *in vivo* experiments show the Cr/CeO_2_ nanozyme can promote wound healing and reduce neuroinflammation of mice following brain trauma. The catalytic patch based on nanozyme provides a noninvasive topical treatment route for TBI as well as other traumas diseases.

**Conclusions:** The catalytic patch based on nanozyme provides a noninvasive topical treatment route for TBI as well as other traumas diseases.

## Introduction

Traumatic brain injury (TBI), one of the most common neurological diseases leading to disability and death, is caused by linear or rotational forces in the brain, producing large amounts of reactive oxygen and nitrogen species (RONS) 1-4. Subsequently, excessive free radicals diffuse into neuronal cells, and then oxidative stress induces inflammation of neuronal cells around the injured position 5-7. Meanwhile, with increasing injury time, the secondary damage can further produce superoxide anion radicals (O_2_^•-^), hydroxyl radicals (^•^OH), peroxynitrite (ONOO^-^), hydrogen peroxide (H_2_O_2_) and nitric oxide (^•^NO) in mitochondrion, which significantly aggravate oxidative damage and cause the continuous brain injury 8-12. Therefore, the efficient clearance of RONS is crucial for the treatment of TBI 13-15.

At present, inorganic nanozymes, such as V_2_O_5_, Fe_3_O_4_, Mn_3_O_4_ and CeO_2_, have attracted widespread attentions due to their good controllability and enzyme-like activities 16-35, exhibiting a favorable prospect for the treatment of TBI. However, catalytic activity of nanozymes is suboptimal and the mimetic enzyme activities are singularized. For example, the V_2_O_5_ nanozyme shows glutathione peroxidase (GPx)-like and peroxidase (POD)-like activity, displays poor performance in simulating superoxide dismutase (SOD) and catalase (CAT) 36-38. The Fe_3_O_4_ nanozyme also performs excellent POD-like activity, but low efficiency to SOD-like, CAT-like and GPx-like activities 39. Similarly, it was reported that CeO_2_ nanozyme possesses enzyme mimetic properties, including SOD and CAT, due to its dual oxidation states can serve as an effective catalyst 40-41, but its GPx-like activity has rarely been mentioned before. Therefore, it is still necessary to further improve the catalytic activity of CeO_2_ with multienzyme-like property for biomedical applications at ultralow treatment dose. Additionally, previous works have suggested that the ratio of Ce^3+^/Ce^4+^ is one of the most important roles for catalytic efficiency, and the high ratio of Ce^3+^/Ce^4+^ will induce the high catalytic activity due to increasing reduced state 42-44. The Zirconia incorporated CeO_2_ induce a higher Ce^3+^/Ce^4+^ ratio and faster conversion from Ce^4+^ to Ce^3+^ than those CeO_2_, leading to significant improvement of RONS scavenging performance 45. Thus, it is highly desirable to improve the catalytic property of CeO_2_ via doping engineering and develop the nanozyme patch for TBI treatment 46.

**Scheme 1:**
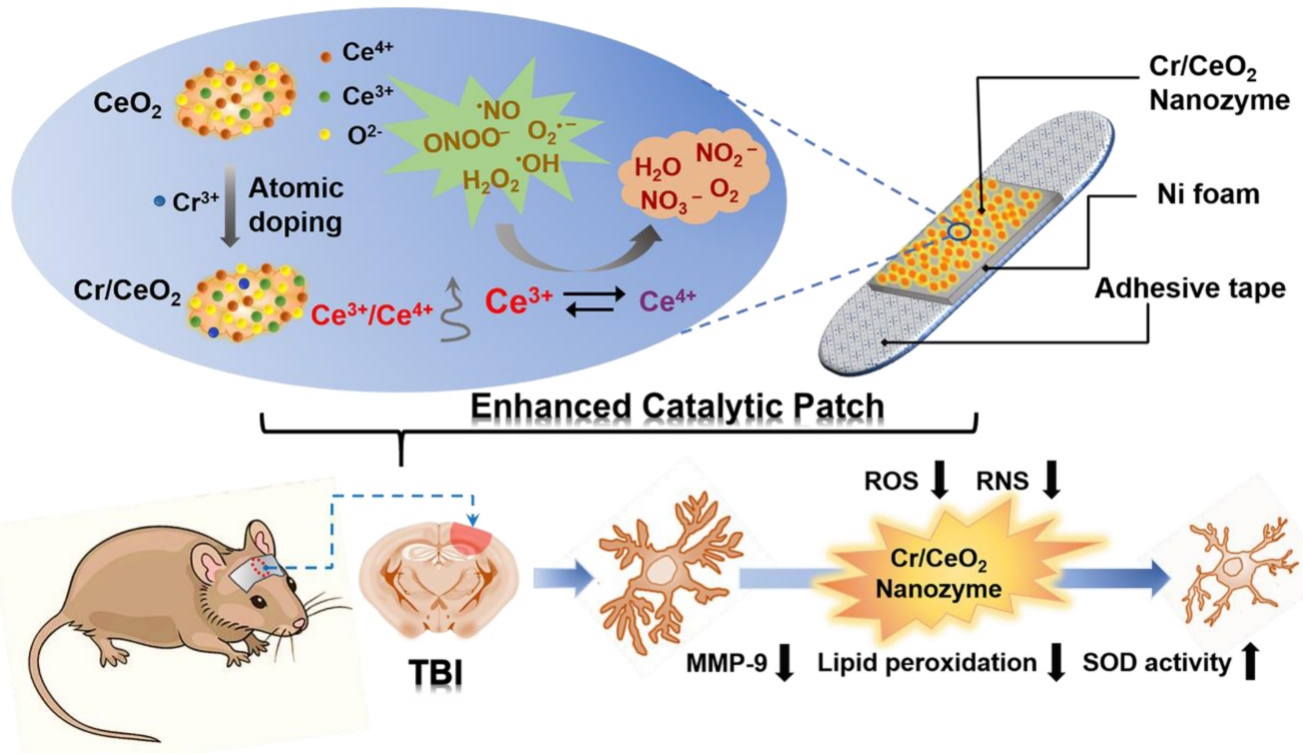
Noninvasive intervention mechanism for brain trauma via enhanced catalytic patch with redox Cr/CeO_2_ nanozyme.

Herein, we developed a catalytic patch based on redox Cr-doped CeO_2_ (Cr/CeO_2_) nanozyme for noninvasive TBI treatment as shwon in **Scheme 1**. The Cr/CeO_2_ nanozyme was synthesized by doping Cr^3+^ ions to improve the ratio of Ce^3+^ to Ce^4+^, thereby improving the catalytic activity. The developed nanozyme displays multienzyme-like property, such as SOD, CAT and GPx, and scavenging activity to free radicals, including ^•^OH, ONOO^-^ and H_2_O_2_, which are remarkably higher than that of undoped CeO_2_. Furthermore, the nanozyme patch can diminish the accumulation of RONS in injured cells, and be helpful for improving wound healing of TBI via reducing the neuroinflammation.

## Methods

### Materials

Cerium(Ⅲ) nitrate hexahydrate (Ce(NO_3_)_3_•6H_2_O, 99.5% metal basis) and Chromic nitrate nonahydrate (Cr(NO_3_)_3_·9H_2_O, 99%) were purchased from Shanghai Macklin Biochemical Co., Ltd. (China). ethylenediaminetetraacetic (EDTA, 99.5%) and citric acid were obtained by Shanghai Aladdin Biochemical Co., Ltd. (China). Ammonium hydroxide (NH_3_•H_2_O, 30% basis) and ethanol were purchased from Jiangtian chemical industry (China). Horseradish peroxidase (HRP) was purchased from Sigma-Aldrich, which was of 300 IU/mg of lyophilized solid. All aqueous solutions in the experiment were using deionized water (Millipore, 18.2 MΩ cm). All compounds used are analytically pure.

### Synthesis of Cr-doped ceria nanozyme (Cr/CeO_2_)

In the work, the monodisperse Cr/CeO_2_ nanozyme with an elliptical shape was prepared by coprecipitation method. Initially, to a 30 mL beaker was added citric acid (0.048 g) and EDTA (0.073 g), followed by deionized water (10 mL). Place the beaker under a high-speed magnetic stirring at 1000 rpm in air. Meanwhile, Cr(NO_3_)_3_•9H_2_O (0.8 g) was dissolved in deionized water (2 mL) to form the aqueous solution of Cr(NO_3_)_3_ (1 mmol/mL). Then Ce(NO_3_)_3_•6H_2_O (0.7814 g) and Cr(NO_3_)_3_ aqueous solution (200 µL was added to the beaker. The clear solution turned a dark blue in color. Subsequently, 28%-30% ammonium hydroxide (260 µL) was added under vigorous stirring. The solution was stirred for 2 h at room temperature to form a cloudy solid-liquid mixture. Then, the precipitate in the mixture was collected by centrifugation for 5 min and washed several times with distilled water and ethanol, respectively, to remove the unreacted reactants and impurities. Afterwards, dried at 120 °C for 12 h in oven. Eventually, the obtained dark blue powder was calcined at 550 °C for 4 h to form dark brown powder, namely Cr/CeO_2_. In addition, Cr(NO_3_)_3_•9H_2_O was replaced by AgNO_3_, Co(NO_3_)_2_•6H_2_O, RhCl_3_•3H_2_O, Mn(NO_3_)_2_•4H_2_O, PdCl_2_ or Ni(NO_3_)_2_•6H_2_O to synthesize CeO_2_ nanozymes doped with different metal elements, which were named by M/CeO_2_. The prepared M/CeO_2_ nanozymes were used in the following characterization and biomedical applications.

### Preparation of catalytic patch with Cr/CeO_2_ nanozyme

First, the porous nickel foams were treated with dilute hydrochloric acid, acetone, ethanol, and deionized water for 10 min, respectively. Then, the purified Cr/CeO_2_ nanozyme were dispersed in water by ultrasound. The nickel foams were soaked in the solution containing nanoparticles in a 120 °C oven for 12 h. Finally, the nickel foams loaded with nanoparticles were annealed at 550 °C for 4 h. The catalytic patch was affixed to a viscous material for later use.

### Characterization

TEM and X-ray spectroscopy (EDX) elemental mapping images of as-prepared Cr/CeO_2_ nanozyme sample were taken on a JEOL-2000EX transmission electron micrograph, with accelerating voltage of 200 kV. X-ray diffraction technology was used to investigate the crystal structure of XRD spectra were conducted on X-ray Powder diffractometer (D/MAX-2500, Rigaku, Japan) with Cu Kα radiation. The valence states of elements measured by X-ray photoelectron spectrometer (PHI 1600, Perkin Elmer, USA) with the Al Kα excitation. Optical properties were measured characterized by ultraviolet-visible (UV-vis) absorption spectroscopy operating at a Varian Lambda 750 UV-VIS NIR instrument (Shimadzu, Japan) while the cuvette was utilized to contain Cr/CeO_2_ nanozyme hydration solutions.

### Multienzyme-like activity evaluation

This experiments were carried out by a modified procedure according to previous reports 47. The 3,3,5',5'-tetraMethyl benzidine (TMB) substrate color development kit (ELISA) was used to detect the POD-like activity of Cr/CeO_2_ nanozyme. According to the instructions, TMB buffer, TMB color developing solution and TMB oxidizing agent are mixed at a ratio of 5000:5000:4 to prepare the TMB color developing working solution. The TMB buffer consists of an acetate buffer with a pH of 6. In 96-well plates, the Cr/CeO_2_ nanoenzyme (20 μL) with different concentrations (0.1, 0.4, 0.8, 1.5, 3 and 6 mg/mL)were added, followed by TMB color developing working solution (200 μL) and incubated for 30 min at room temperature in the dark. At the same time, the evolution of absorbance at 630 nm with time was measured though a microplate spectrophotometer (CMax Plus, Molecular Devices). Eventually, the reaction solution turned blue and photographed for recording. In addition, Cr/CeO_2_ nanoenzyme replaced by deionized water (20 µL) was used as blank control.

The SOD-like activity of CeO_2_ and Cr/CeO_2_ nanozyme were studied by classical nitro-blue tetrazolium (NBT) chromogenic method. According to experimental scheme, a SOD working solution were prepared by mixing SOD detection buffer (250 µL), NBT color developing solution (75 µL) and NBT enzyme solution (75 µL). Then, CeO_2_ (3 mg/mL, 50 µL) or Cr/CeO_2_ (3 mg/mL, 50 µL) nanozymes were added to the SOD working solution (400 µL), respectively. Subsequently, the reaction start solution (50 µL) was added and illuminated for 10 min. The absorbance of formazan at 560nm with reaction time was detected by UV-vis spectrophotometer, indicating the superoxide scavenging ability. In addition, the difference in absorbance of reduced formazan was collected by varying the concentration of Cr/CeO_2_ nanozyme (0-6 mg/mL). Finally, the SOD-like activity of the nanozyme, the clearance rate of superoxide and the concentration-dependent SOD-like activity were calculated by colorimetric analysis.

The CAT-like activity of CeO_2_ and Cr/CeO_2_ nanozymes were evaluated by measuring the decrease in absorbance of H_2_O_2_ at 240 nm. First, the absorption spectrum of H_2_O_2_ solution (50 µmol/L) is measured by UV-vis spectrophotometer. Then, CeO_2_ (3 mg/mL, 10 µL) or Cr/CeO_2_ nanozymes (3 mg/mL, 10 µL) were mixed with H_2_O_2_ solution (50 µmol/L, 190 µL) and incubated for 2 h in the dark. Then, the mixed solutions with generated bubbles were photographed and the absorption spectra were obtained, respectively. Finally, the clearance rate of H_2_O_2_ was quantified.

The GPx-like activity of Cr/CeO_2_ nanozyme was determined adopting the classical Glutathione reductase (GR)-coupled assay by detecting the decrease in absorbance of reduced nicotinamide adenine dinucleotide phosphate (NADPH) at 340 nm. In a assay, the reaction solution is mixed by Cr/CeO_2_ nanozyme (3 mg/mL, 30 µL), reduced glutathione (GSH) (2 mmol/L, 50 µL), GR (17 units, 50 µL), NADPH (200 µmol/L, 50 µL), H_2_O_2_ (1 mmol/L, 10 µL) and pH 7.4 phosphate buffer (310 µL). Then, the reaction was monitored during 10 min on a UV-vis spectrophotometer operating under kinetic mode. Next, the concentrations-dependent GPx-like activity of Cr/CeO_2_ nanozyme was tested by varying the doses of Cr/CeO_2_ nanozyme (0-70 µL), while other reactants are fixed. The GPx-like activity of CeO_2_ enzyme was used as a control in the experiment. Finally, the corresponding reaction rate was calculated.

### Kinetic analysis

Steady-kinetic measurements were conducted by monitoring the absorbance change at 652 nm on a microplate reader 48. The assay was performed using 50 µg/mL Cr/CeO_2_ nanozyme or 0.02 ng/mL HRP in a reaction volume of 200 µL sodium acetate buffer solution (pH 4) with 800 µM TMB and 50 mM H_2_O_2_ as substrates. When the 800 µM TMB as substrate is fixed, the concentration of H_2_O_2_ is varied: 0, 0.025, 0.25, 0.5 1, 2.5, 5, 25 and 50 mM. On the contrary, the 50 mM H_2_O_2_ as substrate is fixed, the concentration of TMB is varied: 0, 12.5, 25, 50, 100, 200, 400 and 800 µM. Then, maximum initial velocity (*V_max_*) and Michaelis-Menten constant (*K_m_*) were calculated using Lineweaver-Burk plots of the double reciprocal of the Michaelis-Menten equation, 1/ν=*K_m_*/*V_max_*•(1/[S]+1/*K_m_*), where v is the initial velocity and [S] is the concentration of substrate.

### RONS scavenging capacities of Cr/CeO_2_ nanozyme

The ^•^OH scavenging capacity of Cr/CeO_2_ nanozyme was qualitatively tested by electron spin resonance (ESR) spectroscopy (Bruker EMX plus, Germany). In the experiment, 5-tert-Butoxycarbonyl-5-methyl-1-pyrroline N-oxide (BMPO) (1 mg) was dissolved in buffer (195 µL, potential of hydrogen: 7.2-7.4) and H_2_O_2_ (5 µL, 40 mmol/L) was added. The mixture was irradiated under a UV lamp for 5 min to generate ^•^OH, of which BMPO was a capture agent. Then, comparing the intensity of the ESR spectrum with or without the presence of CeO_2_ or Cr/CeO_2_ nanozyme (6 mg/mL, 10 µL).

The O_2_^•-^ scavenging capacity is recorded by ESR spectroscopy as described above. O_2_^•-^ is derived from KO_2_ with ultraviolet light treatment and accurately tapped by BMPO. The specific operation was as follows: 18-crown-6 (1 mg) was dissolved in a medium solution of DMSO (10.81 mL) to form an 18-crown-6 solution (3.5 mmol/L), which was stored in a refrigerator. KO_2_ (2 mg) is fully dissolved in the 18-crown-6 solution (562.5 µL, 3.5 mmol/L) under ultrasound, followed by the addition of BMPO (25 mmol/L). Subsequently, the mixture was exposed to UV light for 5 min to produce O_2_^•-^, and ESR spectra were collected as a blank control in time. The ESR spectra were again measured and analyzed after adding CeO_2_ or Cr/CeO_2_ nanozymes (6 mg/mL, 10 µL), respectively.

^•^NO radicals are obtained by a combination of Carboxy-PTIO and S-nitroso-N-acetyl-penicillamine (SNAP), while EPR spectra, as above-mentioned, are applied to assess the ^•^NO scavenging ability. In a typical trial, the first step is to dilute the Carboxy-PTIO (250 mmol/L) to 10 µmol/L with phosphate-buffered saline (PBS) and disperse SNAP (1 mg) in PBS (900 µL) to acquire SNAP solution (5 mmol/L),. Thereafter, the final solution consisted of the Carboxy-PTIO solution (190 μL, 10 mmol/L) and the SNAP solution (10 µL, 5 mmol/L), and the reaction was carried out for 15 min to engender ^•^NO. The 7-peak signals were examined through the ESR spectra. Eventually, add CeO_2_ or Cr/CeO_2_ nanozymes (6 mg/mL, 10 µL) to measure again and appraise the difference in ESR spectra was with and without nanozymes.

Due to ONOO^-^ has a significant absorption peak at 302 nm, the ONOO^-^ scavenging ability was represented by detecting the change of the absorption spectrum in the range of 200-600 nm on a UV-vis spectrophotometer. First, HCl (1 mol/L), NaOH (1.5 mol/L), NaNO_2_ (50 mmol/L), and H_2_O_2_ (25 mmol/L) aqueous solutions were prepared. Then, 10 mL of NaNO_2_ solution and 10 mL of H_2_O_2_ were blended and stirred for 3 min, followed by 5 mL of HCl solution and rapidly injected 5 mL of NaOH solution. It is especially critical that the entire formulation process is carried out in an ice bath. As a result, the solution turned pale yellow to indicate the production of the ONOO^-^. Afterwards, UV-vis spectrophotometer is selected to quantitatively dilute the concentration of ONOO^-^, i.e., the absorbance at 302 nm (A302) is about 0.7. Thereafter, CeO_2_ or Cr/CeO_2_ nanozymes (3 mg/mL, 10 µL) were added to the weak solution (190 µL) to observe the decrease of A302 with the reaction time, and the reduction in ONOO^-^ itself was measured as the background value in the same time. Finally, the actual value of ONOO^-^ elimination under the action of nanozymes was calculated by deducting the background value.

For 1,1-diphenyl-2-picrylhydrazyl (DPPH) scavenging capacity, the absorption spectrum was selected to record at 300-700 nm due to its characteristic peak at 535 nm. In a typical assay, DPPH is dissolved in DMSO as a stock solution and then diluted to 25 µmol/L with deionized water with an absorbance at 535 nm (A535) of approximately 0.42. The A535 was measured by the addition of DPPH solution (195 µL) in the cuvette, and the absorption spectrum was measured after adding CeO_2_ or Cr/CeO_2_ nanozymes (6 mg/mL, 5 µL) at intervals of 10 min. The time dependence of the diminishable on A535 was evaluated with the addition of nanozyme, which is the DPPH scavenging capacity of the nanozyme.

### Cell toxicity

In a 96-well plate, HT22 cells were cultured with Dulbecco's modified eagle medium (DMEM) at 37 ℃ (about 4000 cells per well). After 12 h of growth, a series of CeO_2_ or Cr/CeO_2_ nanozyme solutions were added into each well at a dose of 6 to 100 µg/mL, respectively. The cells were then incubated for another 24 h and the cell viabilities were measured by MTT assay.

### Cell survival

HT22 cells were seeded in 96-well plates (approximately 2000 per well) and grown for 12 h in a 37-read incubator. A series of CeO_2_ or Cr/CeO_2_ nanozyme solutions at a dose of 6 to 100 µg/mL were added into wells and maintained for 24 ho to promote cell uptake. Then, the cells were incubated for another 24 h under stimulation with lipopolysaccharide (LPS) solutions (0.5 mg/mL, respectively). Finally, the corresponding cell viabilities were quantified by MTT assay.

### *In vitro* free radical detection

HT22 cells were seeded into 6-well plates at 15,000 cells per well and cultured until fully adherent. First, the DMEM containing CeO_2_ nanozyme (25 µg/mL) or Cr/CeO_2_ nanozyme (25 µg/mL) was added into each well, followed by LPS (0.5 mg/mL). After 6 h of incubation, DCFH-DA ((S0033S, Beyotime), DHE (S0063, Beyotime) or DAF-FM DA (S0019, Beyotime) reagents were added into the 6-well culture plates to detect cellular total ROS, O_2_^•-^ and ^•^NO levels. After a period of time (DCFH-DA, DHE and DAF-FM DA correspond to reaction times of 20, 30 and 20 min, respectively), the plate was washed three times with PBS to stop the reaction. The CeO_2_ nanozyme was used as a control in the experiment. Finally, cell images were captured using a fluorescence microscope (EVOS, AMG) and quantitative analysis was performed by flow cytometry (BD Accuri^TM^ C6). The difference in fluorescence intensity was compared to analyze the level of free radicals in cells.

### Traumatic brain injury (TBI)

C57 BL/6 male mice at 8-10 weeks age with a body weight of 21-23 g were selected to establish TBI model with fluid percussion injury. Each mouse was anesthetized by intraperitoneal injection of 0.2 µL of 10% chloral hydrate. After anesthesia, the head of the mouse was depilated and the skin was cut to separate the meninges from the skull. Then, the head of the mouse was fixed on a stereo azimuth instrument, and a nearly circular opening having a diameter of 5 mm in the right brain was cut with a dental drill. Next, a craniocerebral instrument was used to hit the round hole in the right brain of mouse. During all animal experiments, the specific trauma was made by opening the bone window with a fluid percussion injury (1.7 ± 0.1 atm) for moderate TBI models. Subsequently, the brain injured mice were randomly divided into three groups: TBI mice without treatment (TBI), TBI mice with CeO_2_ nanozyme patch treatment (TBI+CeO_2_), TBI mice with Cr/CeO_2_ nanozyme patch treatment (TBI+Cr/CeO_2_), photographs of the wound were taken once every two days. Finally, a catalytic patch loaded with CeO_2_ or Cr/CeO_2_ nanozymes was adhered t o the brain of mice in TBI+CeO_2_ or TBI+Cr/CeO_2_ groups and replaced every other day.

In addition, we further investigated the concentration and diffusion depth of Cr/CeO_2_ nanozyme released from the patch into brain tissue. Taking the injury location as the starting point, brain slices were made each 2 mm along the longitudinal direction, and then the concentration of Cr/CeO_2_ nanozyme in each section was measured by the inductively coupled plasma mass spectroscopy (ICP-MS).

### Immunofluorescence

Mice in the normal control, TBI, TBI+CeO_2_, and TBI+Cr/CeO_2_ groups were perfused, and the brains were removed and fixed in 4% paraformaldehyde. Then, the brain tissue was made into paraffin slices. The slices were stained by the following procedures: dewaxing in xylene, dehydration in gradient ethanol (100%, 95%, 80%, and 70%), antigen retrieval in sodium citrate or EDTA solutions, addition of primary antibody overnight at 4 ℃, incubation with goat anti rabbit IgG 488 or 594 secondary antibody for 1 h, staining of nuclei with DAPI for 5 min and sealing with fluorescence quencher. For primary antibody, anti-GFAP, Iba1 and matrix metalloproteinase-9 (MMP-9) (Proteintech) were used to stain astrocytes, microglia and MMP-9, respectively. Notably, the staining process was carried out in the dark. Finally, the stained images were acquired by fluorescence microscope (EVOS, AMG) and immunofluorescence analysis was performed by Image J software.

### *In vivo* oxidative stress

Mice were perfused with normal saline on 12 and 26 days after TBI and brains were taken. SOD activity was evaluated according to the experimental protocol in the Total Superoxide Dismutase Assay Kit with WST-8 (Beyotime, S0101). Brain tissue and SOD sample preparation solution were added into centrifuge tube at a mass ratio of 1:10, and homogenized in ice bath. The brain homogenate was centrifuged and the supernatant was used as a sample to be tested. Meanwhile, the SOD standard was diluted to the following series concentrations: 1, 2, 5, 10, 20, 50 and 100 U/mL, for making a standard curve. Then, WST-8 enzyme working solution (160 µL), sample (20 µL) and reaction start solution (20 µL) were sequentially added to the 96-well plate for 30 min, and the absorbance at 450 nm was detected by a multifunctional microplate detector (Synergy HT). The SOD activity of brain tissue was calculated with reference to the standard curve.

The lipid peroxide assay was performed using the Lipid Peroxidation malondialdehyde (MDA) assay kit (Beyotime, S0131). According to the instructions, the brain tissue was homogenized in PBS. At the same time, the standard was diluted to 1, 2, 5, 10, 20 and 50 µmol/L with distilled water to prepare a standard curve. Then, the sample (100 µL) was mixed with MDA test working solution (200 µL) and heated in boiling water for 15 min. The absorbance at 532 nm was measured with a multifunctional microplate detector (Synergy HT). Further, the protein concentration of the sample was measured using BCA Protein Assay Kit (Beyotime, P0010). Finally, the MDA content per unit weight of protein in brain tissue was quantified by the standard curve.

### Morris water maze test

The Morris water maze test was used to assess the differences of spatial learning ability and related memory in the four groups of mice (control, TBI, TBI+CeO_2_, and TBI+Cr/CeO_2_). First, a Morris water maze model was built by injecting 50 cm of water into a large cylindrical water tank, than adding non-toxic white paint to make the water opaque. The water tank is divided into four quadrants, and the platform is hidden about 1 cm below the water surface in the middle of the quadrant I. Video tracking of mice was performed using a camera focused on the tank. The hidden platform training period lasted for 5 days and 4 trials were conducted each day, with the starting sites being located at fixed positions in four different quadrants of the tank wall. In the trial, the mouse found the platform and ended the timing when it was held on the platform for 2 seconds. The maximum duration of the training was 60 seconds. Mice that failed to reach the platform within 60 seconds were directed to the platform and held for 10 seconds. The time to reach the platform, the distance from the platform, and the average swimming speed were recorded during the five days. On the fifth day, the platform was removed and each mouse was allowed to swim for 60 seconds. The spatial learning ability and related memory ability of the mice were evaluated by recording the frequency of the mice through the platform, the average distance to the platform, and the time spent in the first quadrant.

### Hematological test

Mice in TBI, TBI+CeO_2_ and TBI+Cr/CeO_2_ groups were subjected to ocular blood extraction at 12 and 26 days after TBI. The hematological examination of blood was performed in time. Then, the serum was collected by centrifuging for biochemical analysis.

### Statistical analysis

All data in this work were presented as mean ± standard deviation (SD). The statistical analysis was executed by a Student's t test.

## Result and Discussion

### Characterization of Cr/CeO_2_ nanozyme

**Figure 1:**
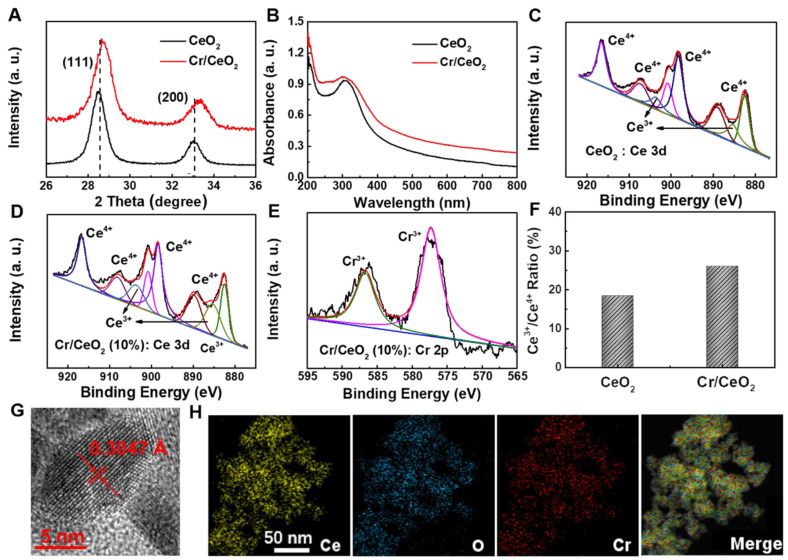
**Materials Properties of Cr/CeO_2_ nanozyme. A)** XRD pattern of CeO_2_ and Cr/CeO_2_ nanozymes. **B)** UV-vis absorption spectra of CeO_2_ and Cr/CeO_2_ nanozymes. **C)** XPS spectra of CeO_2_ nanozymes for Ce 3d. **D, E)** XPS spectra of Cr/CeO_2_ nanozyme for Ce 3d, and Cr 2p, respectively. **F)** Ratio of Ce^3+^ /Ce^4+^ in CeO_2_ and Cr/CeO_2_ nanozymes. **G)** HRTEM image of Cr/CeO_2_ nanozyme. **H)** STEM-EDX mapping images of Cr/CeO_2_ nanozyme.

The Cr/CeO_2_ nanozymes were synthesized by the method of coprecipitation 40, 49. X-ray diffraction (XRD) technology was used to investigate the crystal structure of Cr/CeO_2_. As shown in **Figure 1A**, the (111) and (200) diffraction peaks can be found in both CeO_2_ and Cr/CeO_2_, identifying high crystallinity. Meanwhile, the diffraction angles corresponding to (111) and (200) show the significant shift to a large angle after Cr doping, indicating that the incorporation of Cr^3+^ ions with small atomic radius into CeO_2_ induces lattice distortion. Meanwhile, the UV-vis absorption spectrum in **Figure 1B** shows that a slight red shift in the absorption band edge after Cr doping, suggesting decrease of band gap.

In order to further elucidate detailedly the structural composition, the X-ray photoelectron spectroscopy (XPS) tests of Cr/CeO_2_ and pure CeO_2_ were performed. As shown in **Figure 1C**-**D**, the Ce possesses the dual oxidation state of the +3 and +4 valence states, and Ce^4+^ is located at 907.3, 900.8, 916.6 eV and 889, 898.3, 882.5 eV, ascribed to Ce^4+^ 3d_3/2_ and Ce^4+^ 3d_5/2_, respectively. The peaks at 885.3 and 903.8 eV are assigned to Ce^3+^ 3d_3/2_ and Ce^3+^ 3d_5/2_. In addition, the two peaks at 587 and 577.3 eV in **Figure 1E** correspond to the binding energies of Cr^3+^ 2p_1/2_ and Cr^3+^ 2p_3/2_, revealing the presence of Cr^3+^ ions. Quantative analysis in** Figure 1F** reveals Cr^3+^ incorporation promotes an increase in the ratio of Ce^3+^ to Ce^4+^, as well as production of reduced Cr^3+^ state. Furthermore, the transmission electron microscopy (TEM) and high-resolution TEM (HRTEM) images of Cr/CeO_2_ nanozyme in **Figure S1** and **1G** reveal that the size of nanozyme is about 8~12 nm, and interplanar spacing of the (111) plane of CeO_2_ is slightly reduced from 0.3123 Å to ~0.3047 Å after Cr doping. Additionally, the energy dispersive X-ray spectroscopy (EDX) mapping of Cr/CeO_2_ nanozyme in **Figure 1H** also indicates Cr doping into CeO_2_.

### Optimization of catalytic activity

**Figure 2:**
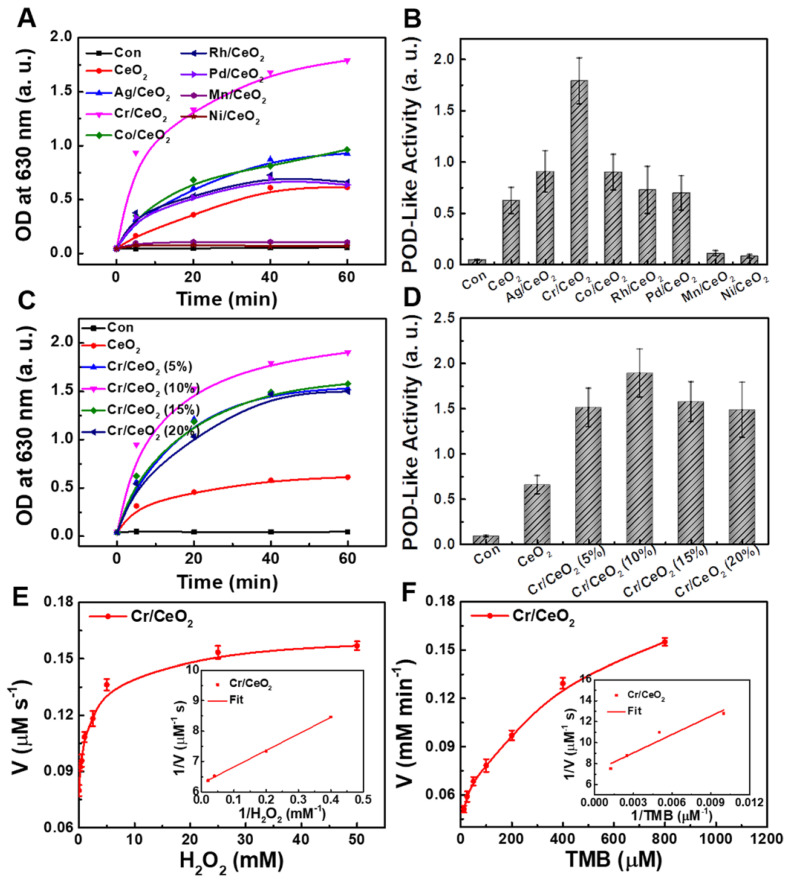
** Optimization of catalytic activity. A)** Time-dependent absorbance of CeO_2_ nanozymes doped with different metal elements at 630 nm in TMB method. **B)** POD-like activity of CeO_2_ nanozymes doped with different metal elements. **C)** Time-dependent absorbance of Cr/CeO_2_ nanozymes with different doping ratios at 630 nm in TMB method. **D)** POD-like activity of Cr/CeO_2_ nanozyme with different doping ratios. Deionized water serves as blank control in all assay, marked as "con". The velocity (v) of the reaction was measured by steady-state kinetic assay using 50 μg/mL Cr/CeO_2_ nanozyme. **E)** The concentration of TMB was 0.8 mM and varied concentration of H_2_O_2_.** F)** The concentration of H_2_O_2_ was 50 mM and varied concentration of TMB. Inset: Double-reciprocal plots of activity of Cr/CeO_2_ nanozyme at a fixed concentration of one substrate versus varying concentration of the second substrate.

To investigate catalytic properties, the differences in catalytic performance of CeO_2_ nanozymes doped with different metal elements (such as Ag, Cr, Co, Rh, Pd, Mn, Ni) were first explored. As show in **Figure 2A**-**B**, the CeO_2_ nanozyme exhibits the best catalytic activity when Cr is doped. Then, a series of Cr/CeO_2_ nanozymes at different doping ratios were synthesized. The CeO_2_ nanozymes with different doping were subjected to a comparative test of POD-like activity. It is noting that Cr/CeO_2_ nanozyme at 10% doping concentration exhibits the best POD-like activity among all samples (**Figure 2C**-**D**). Moreover, the results of XPS spectra show that the Ce^3+^/Ce^4+^ ratio of Cr/CeO_2_ nanozyme is the highest at 10% doping concentration (**Figure S2**), which indicates that the main reason for the enhancement of POD-like activity of Cr/CeO_2_ nanozyme is the increase of Ce^3+^/Ce^4+^ ratio caused by Cr^3+^ doping. Thus, the Cr/CeO_2_ nanozyme at 10% doping concentration was selected as the research object for further exploration. In addition, the typical Michaelis-Menten curves were performed for Cr/CeO_2_ nanozyme (**Figure 2E**-**F**) and HRP (**Figure S3**). To further analyze the catalytic mechanism, steady-state kinetic parameters for the oxidation of TMB in present of H_2_O_2_ were also calculated. The *V_max_* and *K_m_* were obtained using Lineweaver-Burk plot and are shown in **Table 1**. The *K_m_* value of Cr/CeO_2_ nanozyme with H_2_O_2_ as the substrate is in the same order of magnitude as that of HRP. The *K_m_* value of Cr/CeO_2_ nanozyme with TMB as substrate is significantly lower than that of HRP, indicating that Cr/CeO_2_ nanozyme possesses higher affinity to TMB than HRP.

### Multienzyme-like activity evaluation

**Figure 3:**
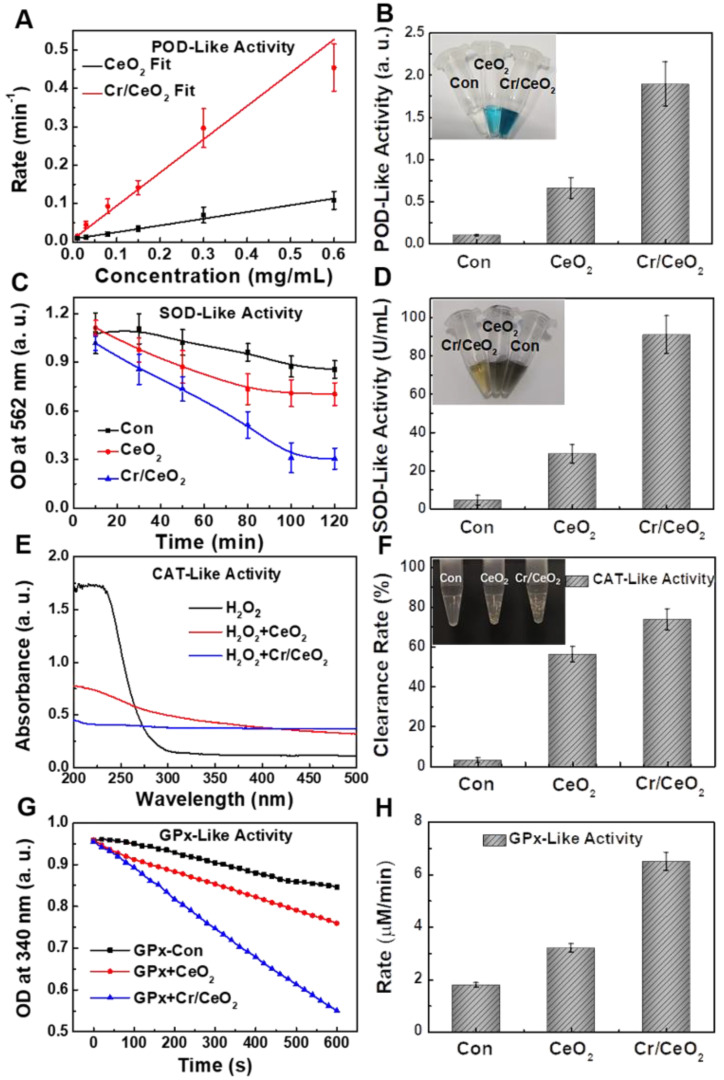
**Multienzyme-like activity of Cr/CeO_2_ nanozyme. A)** Concentration-dependent reaction rate for Cr/CeO_2_ nanozyme in TMB Assay. **B)** POD-like activity of Cr/CeO_2_ nanozyme. Inset: Photo images of final solution after reacting with Cr/CeO_2_ nanozymes for 60 min in TMB method. **C)** Time-dependent absorbance at 562 nm with Cr/CeO_2_ nanozyme in NBT method. **D)** SOD-like activity of Cr/CeO_2_ nanozyme. Inset: Photo images of result solution after reacting with Cr/CeO_2_ nanozymes for 140 min in NBT method. **E)** Absorbance of H_2_O_2_ solution with or without Cr/CeO_2_ nanozyme. **F)** H_2_O_2_ clearance rate of Cr/CeO_2_ nanozyme. Inset: Photo images of H_2_O_2_ solution after reacting with Cr/CeO_2_ nanozyme for 10 min. **G)** GPx-like activity of Cr/CeO_2_ nanozyme by GPx Assay Kit. **H)** Corresponding reaction rate of Cr/CeO_2_ nanozyme was calculated during GPx-like activity assay. Deionized water serves as blank control in all assay, marked as "con".

Based on the results of the catalytic activity in the above exploration, the multienzyme-like property of Cr/CeO_2_ nanozyme was further studied. **Figure 3A-B** reveal the POD-like activity of the Cr/CeO_2_ nanozyme (10% doping) approximately 3-4 times higher than that of CeO_2_, identifying the improvement of catalytic property. In addition, in order to evaluate the stability of Cr/CeO_2_ nanozyme, the Cr/CeO_2_ nanozyme was put in DMEM for one month. Then, the POD-like activity and structure of initial and old Cr/CeO_2_ nanozyme were evaluated. As shown in **Figure S4**, there is no significant difference in the chemical state of Cr between initial Cr/CeO_2_ and old Cr/CeO_2_ nanozyme in performance and structure, which illustrates the excellent stability of Cr/CeO_2_ nanozyme.

Moreover, the SOD-like activity of Cr/CeO_2_ nanozyme was assessed by classical NBT chromogenic method. **Figure 3C** shows that the absorbance at 560 nm changes with reaction time after the uptake of Cr/CeO_2_ nanozyme, indicating efficient SOD-like activity. Quantitative results in **Figure 3D** exhibit the SOD-like activity of Cr/CeO_2_ nanozyme is about 3 times higher than that of CeO_2_. Meanwhile, the SOD-like activity of the Cr/CeO_2_ nanozyme increases almost linearly relative to its concentration (**Figure S5**). Subsequently, the CAT-like activity was determined by comparing the decrease in the absorbance of H_2_O_2_ at 240 nm when CeO_2_ or Cr/CeO_2_ nanozymes were separately added. A significant decrease in the absorbance of H_2_O_2_ with Cr/CeO_2_ is observed, and the clearance rate of H_2_O_2_ is 75%, obviously higher than CeO_2_ (**Figure 3E**-**F**). In addition, the CAT-like activity of Cr/CeO_2_ nanozyme in neutral and alkaline environment is better than the level of acid environment, but comparable to the activity in inflammatory environment (**Figure S6**). Similarly, the GPx-like activity of Cr/CeO_2_ was investigated by detecting the evolution in absorbance of NADPH at 340 nm according to the classical glutathione reductase coupling reaction mechanism. As show in **Figure 3G**-**H**, the GPx-like activity of the Cr/CeO_2_ nanozyme turn a significant enhancement compared to CeO_2_, with a reaction rate of 6.5 µmol L^-1^·min^-1^. **Figure S7** reveals that the reaction rate is also proportional to the concentration of nanozyme, suggesting the good GPx-like activity. In addition, the CAT-like, GPx-like and SOD-like activities of nanozyme with different doping metal elements and different Cr-doping concentrations were also investigated. The results show that the CeO_2_ nanozyme with 10% Cr-doping exhibits the optimal multienzyme-like activity (**Figure S8**). Importantly, the pure CeO_2_ nanozyme mainly exhibits SOD-like and CAT-like enzyme activities, consistent with previous reports in the literature 50-54, while as-obtained Cr/CeO_2_ nanozyme is capable of simulating three major antioxidant enzymes, including SOD, CAT and GPx, due to the incorporation of Cr^3+^ ions. Thereby, Cr/CeO_2_ nanozyme is expected to play an important role in intracellular antioxidants by the synergistic effect of multienzyme-like properties, maintaining intracellular redox homeostasis.

### RONS scavenging capacities of Cr/CeO_2_ nanozyme

**Figure 4:**
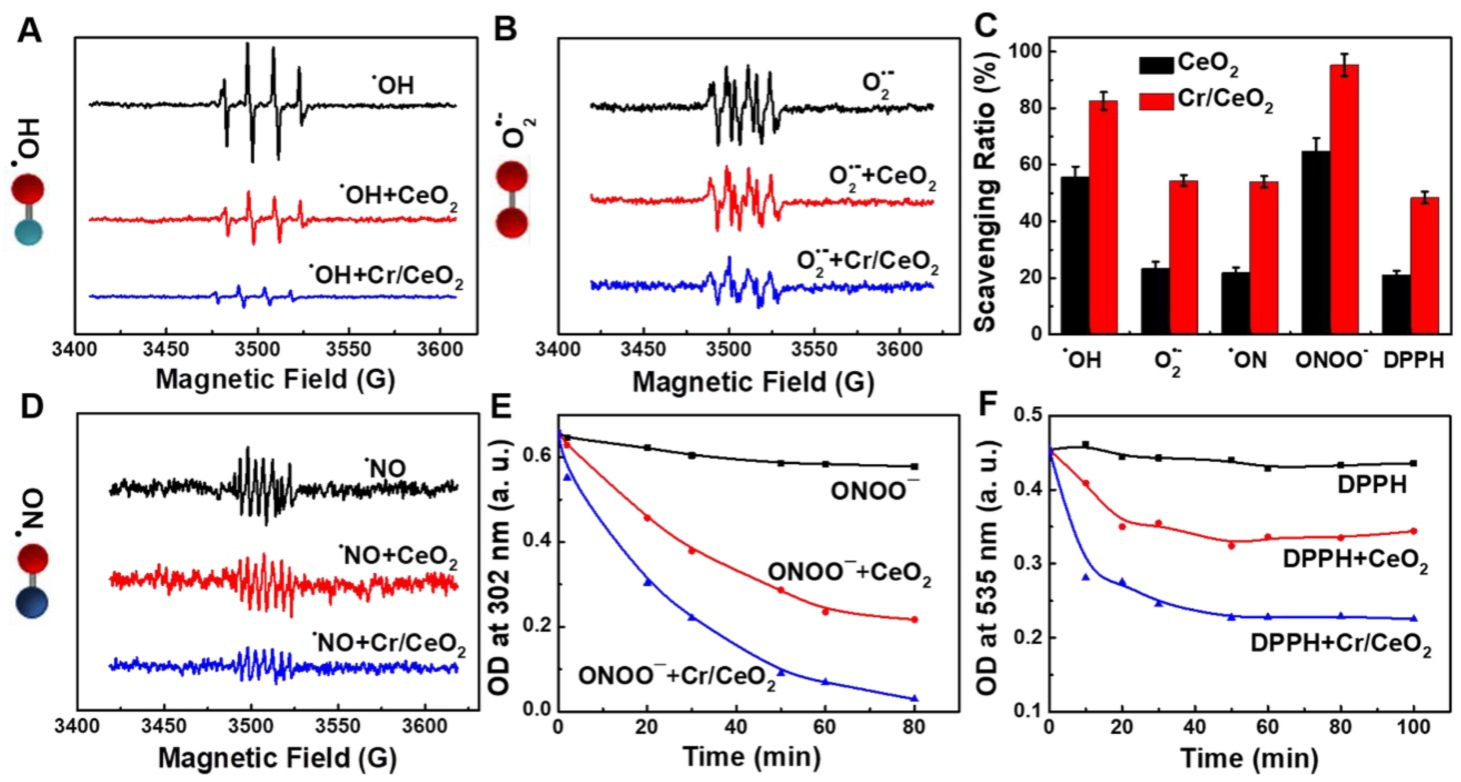
**RONS scavenging selectively of Cr/CeO_2_ nanozyme.** ROS scavenging abilities of the Cr/CeO_2_ nanozyme for **A)** ^•^OH and **B)** O_2_^•-^ tested by ESR, respectively. **C)** Comparison of the ability of Cr/CeO_2_ nanozymes to scavenge free radicals for ^•^OH, O_2_^•-^, ^•^NO, ONOO^-^ and DPPH. RNS scavenging abilities of the Cr/CeO_2_ nanozyme for **D)** ^•^NO, **E)** ONOO^-^ and **F)** DPPH tested by ESR and UV-vis absorption spectra, respectively.

Furthermore, to determine the free radicals scavenging abilities of the Cr/CeO_2_ nanozyme, the ESR spectroscopy was performed. As show in **Figure 4A**-**B**, the ESR signals are significantly reduced after adding Cr/CeO_2_ nanozyme, indicating the scavenging rate to ^•^OH and O_2_^•-^ free radicals. The nanozyme displays the higher sensitivity to ^•^OH than O_2_^•-^ at same concentration (**Figure 4C**). For reactive nitrogen species (RNS) scavenging, the total RNS level has improved, but it remains to be enhanced. The Cr/CeO_2_ nanozyme prefers to clean up ONOO^-^ with a clearance rate as high as 93%, and the clearing of ^•^NO is only 57%, but still obviously higher than that of CeO_2_ (**Figure 4D**-**E**). Moreover, the results reveal that the ONOO^-^-scavenging performance of Cr/CeO_2_ nanozyme in neutral environments is better than that of alkaline environments (**Figure S9**). The DPPH radical acts as a very stable nitrogen-centered free radical due to its specific structure with three benzene rings. The clearance rate of Cr/CeO_2_ nanozyme against DPPH is improved to 50%, which is 2.5 times that of undoped CeO_2_ (**Figure 4F**). Therefore, it has been demonstrated that the Cr doping can enhance the free radicals scavenging ability to multiple RONS, which is vital for preventing oxidative damage in biosystem. In particular, the ability of Cr/CeO_2_ nanozyme to selectively purify RNS, an important signaling molecules in the brain, was rarely reported before 55-57. This result makes it highly potential for the treatment of brain injury.

### *In vitro* cytotoxicity and free radical detection

**Figure 5:**
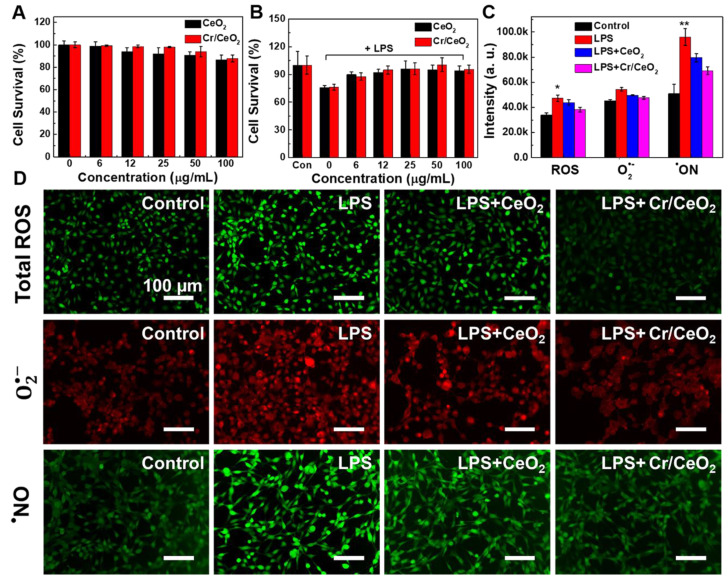
*** In vitro* treatment of Cr/CeO_2_ nanozyme**. **A)** Cell survival of HT22 cells treated with different concentrations of Cr/CeO_2_ nanozyme measured by MTT assay. **B)** Cell survival of LPS induced HT22 cells treated with different concentrations of Cr/CeO_2_ nanozyme measured by MTT assay. **C)** The corresponding quantitative analysis by a flow cytometer and **D)** fluorescence images of total ROS, O_2_^•-^ and ^•^NO levels in HT22 cells induced by LPS in the presence or absence of Cr/CeO_2_ nanozyme were measured using DCFH-DA, DHE and DAF-FM DA probes, respectively. Asterisks indicate significant differences (*P < 0.05, **P < 0.01, ***P < 0.001) as compared with the control group, analyzed by a Student's t test.

Inspired by the above work, the survial rate of injured neuron cells after nanozyme treatment was detected firstly to evaluate the *in vitro* free radicals scavenging abilities of Cr/CeO_2_ nanozyme. The results show high cellar viability to HT22 cells within the nanozyme doses of 100 µg/mL (**Figure 5A**), illustrating low cytotoxicity of Cr/CeO_2_ nanozyme. Then, the LPS were used to stimulate the cells, and the survival rate of HT22 cells decreased to 80%, indicating obvious toxicity after 24 h uptake. However, the viabilities of injured cells are improved gradually under the treatment of Cr/CeO_2_ nanozymes with different concentrations (**Figure 5B**). Meanwhile, the total ROS, O_2_^•-^ and ^•^NO levels induced by LPS in the presence or absence of Cr/CeO_2_ nanozyme treatment were monitored using DCFH-DA, DHE and DAF-FM DA probes. As show in **Figure 5C-D**, both flow cytometry and fluorescence images exhibit the same trend for RONS. For the total ROS, it can be seen that fluorescence intensity of injured cells enhances after incubation of LPS, indicating RONS production. The fluorescence intensity decreases after nanozyme treatment, and the reduction of Cr/CeO_2_ nanozyme is more significant than that of undoped CeO_2_. As for O_2_^•-^ and ^•^NO, the change of fluorescence intensity is basically consistent with ROS. The results suggest that Cr/CeO_2_ nanozyme can remove RONS and prevent cells from the oxidative damage induced by LPS. Under the stimulation of LPS, intracellular RONS levels are rapidly elevated, resulting in damage to DNA, protein and lipids 58-59. Crucially, it is proved that Cr/CeO_2_ nanozyme can improve the survival rate of neuron cells by eliminating oxidative stress induced by RONS.

### TBI treatment by Cr/CeO_2_ nanozyme catalytic patch

**Figure 6:**
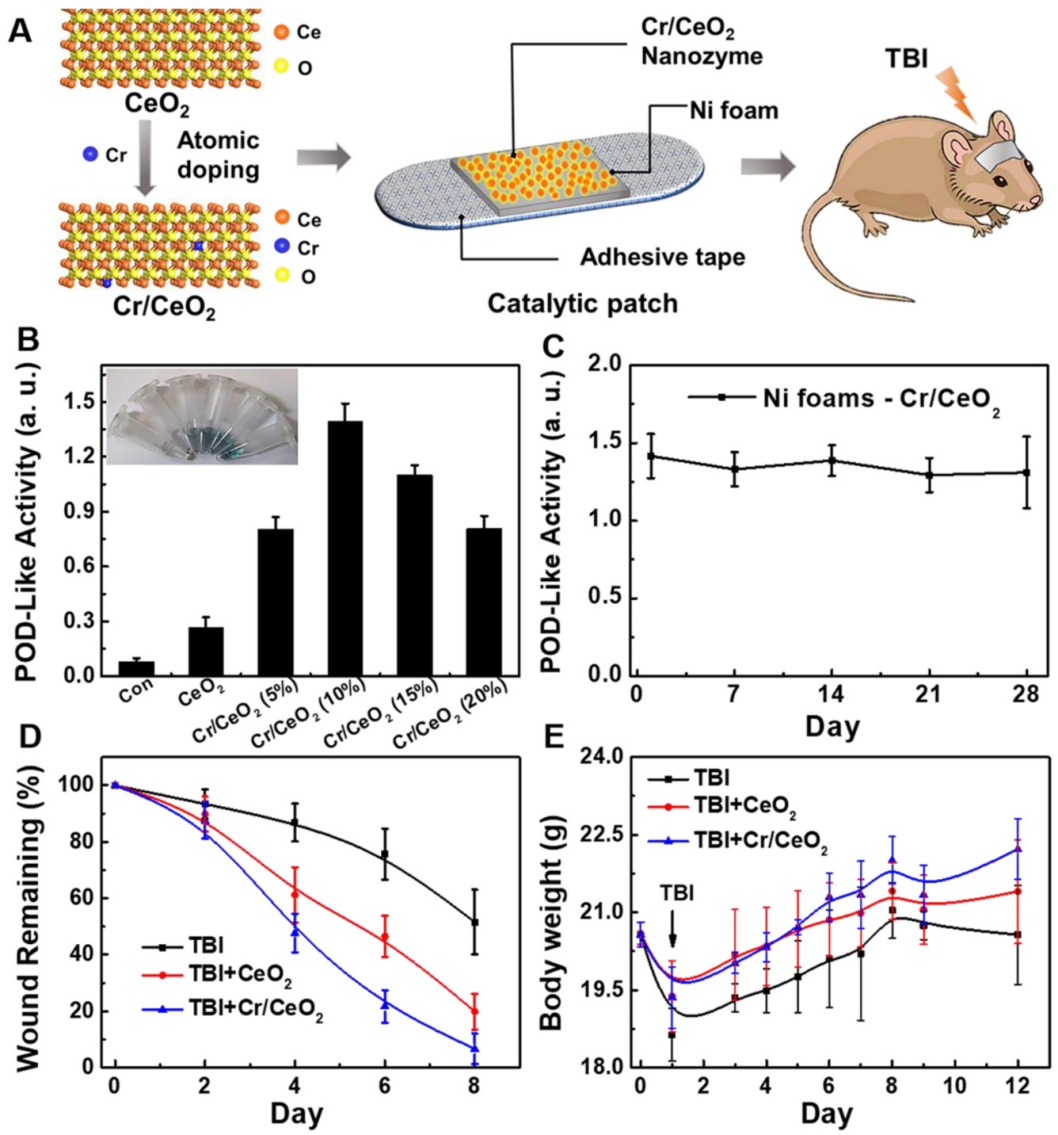
** Design of nanozyme patch and TBI treatment. A)** Design of catalytic patch base on Cr/CeO_2_ nanozyme for TBI treatment. **B)** POD-like activity of catalytic patch base on Cr/CeO_2_ nanozyme with different doping. **C)** Stability of Cr/CeO_2_ nanozyme patch at 10% doping concentration. **D)** Wound length of mice in the presence or absence of nanozyme patch treatment after TBI. **E)** Body weight of mice with or without nanozyme patch treatment after TBI.

The multienzyme-like activity of Cr/CeO_2_ nanozyme *in vitro* encourage us to explore the *in vivo* TBI treatment of nanozymes in noninvasive route. In order to minimize toxicity and side effects, a Cr/CeO_2_ nanozyme patch is developed to treat TBI. As show in **Figure 6A**, catalytic patch is based on Cr/CeO_2_ nanozyme, and the porous nickel foams as supporting materials. **Figure 6B** reveals the Cr/CeO_2_ nanozyme still possesses high catalytic activity after forming nanozyme patch, and the antioxidant properties of the catalytic patch did not diminish after one month (**Figure 6C**), proving good stability. The C57 mice aged 6-8 weeks were established in TBI model, and were randomly divided into three groups (*n* = 13 in each group). The wound sizes of mice in all three groups were measured every other day. The wounds of mice with Cr/CeO_2_ nanozyme patch treatment show almost complete healing on the 8th day, and wounds of mice in TBI group exhibit slow recovery (**Figure 6D** and **S10**). Besides, the body weights of mice in TBI+Cr/CeO_2_ group also grow faster than that of mice in TBI group (**Figure 6E**). Furthermore, the concentration and diffusion depth of Cr/CeO_2_ nanozyme released from the patch into brain tissue through the injured site were monitored by ICP-MS. The results show that the Cr/CeO_2_ nanozyme released from the patch and spread into the brain tissue through the injury location within 6 hours after brain injury (**Figure S11**). After TBI injury, the blood-brain barrier (BBB) is in the open state for a short time, and the Cr/CeO_2_ nanozyme released from the patch can pass through the BBB and spread into the brain tissue. Therefore, the topical treatment based on Cr/CeO_2_ nanozyme patch accelerates wound healing, which is in agreement with earlier report that oxidative stress in the microenvironment of wound site is alleviated by inhibiting ROS levels 60-62, and relieves the effects of brain injury in body weight. It is speculated that these results potentially have a potential therapeutic effects on the recovery of brain tissue levels in TBI.

**Figure 7:**
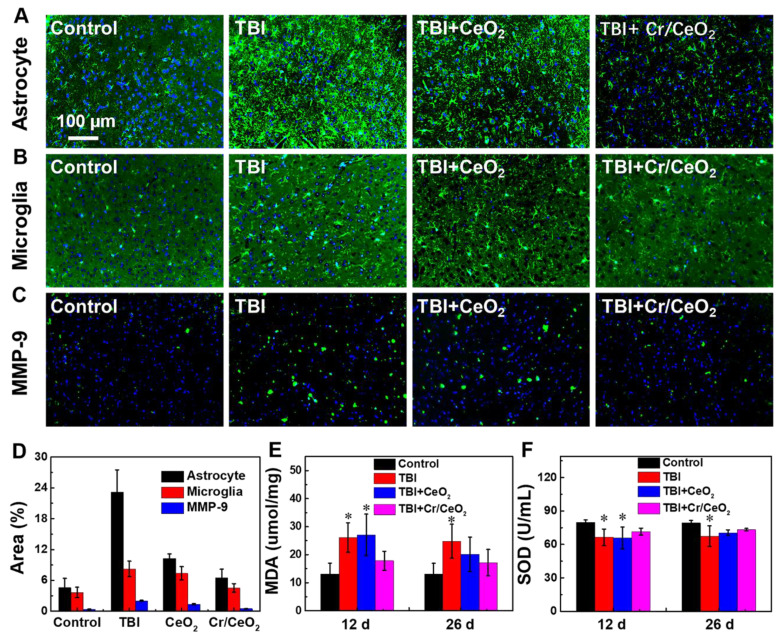
***In vivo* treatment of Cr/CeO_2_ nanozyme**. **A-C)** Immunostaining image of astrocytes, microglia and MMP-9 of the brain sections at 12 days after injury were detected using anti-GFAP, anti-Iba-1 and anti-MMP-9, respectively. **D)** Corresponding analysis of astrocytes, microglia and MMP-9 based on the immunostaining image were calculated by ImageJ software. **E)** MDA levels in brain tissue was tested at 12 and 26 days after injury. **F)** SOD activity was quantified at 12 and 26 days after injury. All tests were performed on four groups: healthy control, TBI, TBI+CeO_2_ and TBI+Cr/CeO_2_ nanozyme patch. Asterisks indicate significant differences (*P < 0.05, **P < 0.01, ***P < 0.001) as compared with the control group, analyzed by a Student's t test.

To further understand the effect of Cr/CeO_2_ nanozyme patch on cerebral oxidative stress-induced neuronal responses, immunofluorescence staining of the brain sections was carried out to observe the difference among the brain injured mice with or without patch treatment. The morphology and number of astrocytes and microglia in **Figure 7** are analyzed. When mice are subjected to brain injury, a large number of astrocytes and microglia are activated by the acute immune response, and the treat of nanozyme patch can remarkably decrease the neuroinflammation (**Figure 7A**-**B**). Similarly, MMP-9, a pro-inflammatory factor, is also a biomarker for testing inflammation in the brain. The MMP-9 shows high expression level following TBI, but it reduces sharply after nanozyme patch treatment (**Figure 7C**). Quantitative analyses of the difference in immunofluorescence also confirm that the treatment of Cr/CeO_2_ nanozyme patch can improve the neurons loss in brain tissue following TBI, alleviating neuroinflammation 63-65 (**Figure 7D**). Additionally, elevated levels of RONS cause an increase in lipid peroxidation and inhibit the activity of SOD in the brain. The results reveal that the lipid peroxidation from the brain tissue can be increased and SOD activity can be decreased within 12 days after TBI. Whereas the treatment with nanozyme patch reduces lipid peroxidation and increases the SOD activity (**Figure 7E-F**). Thereby, it is further confirmed that the nanozyme patch-based topical treatment is an effective route for alleviating excessive oxidative stress in the brain and improving physiological environment of nerve cells.

### Behavior test

**Figure 8:**
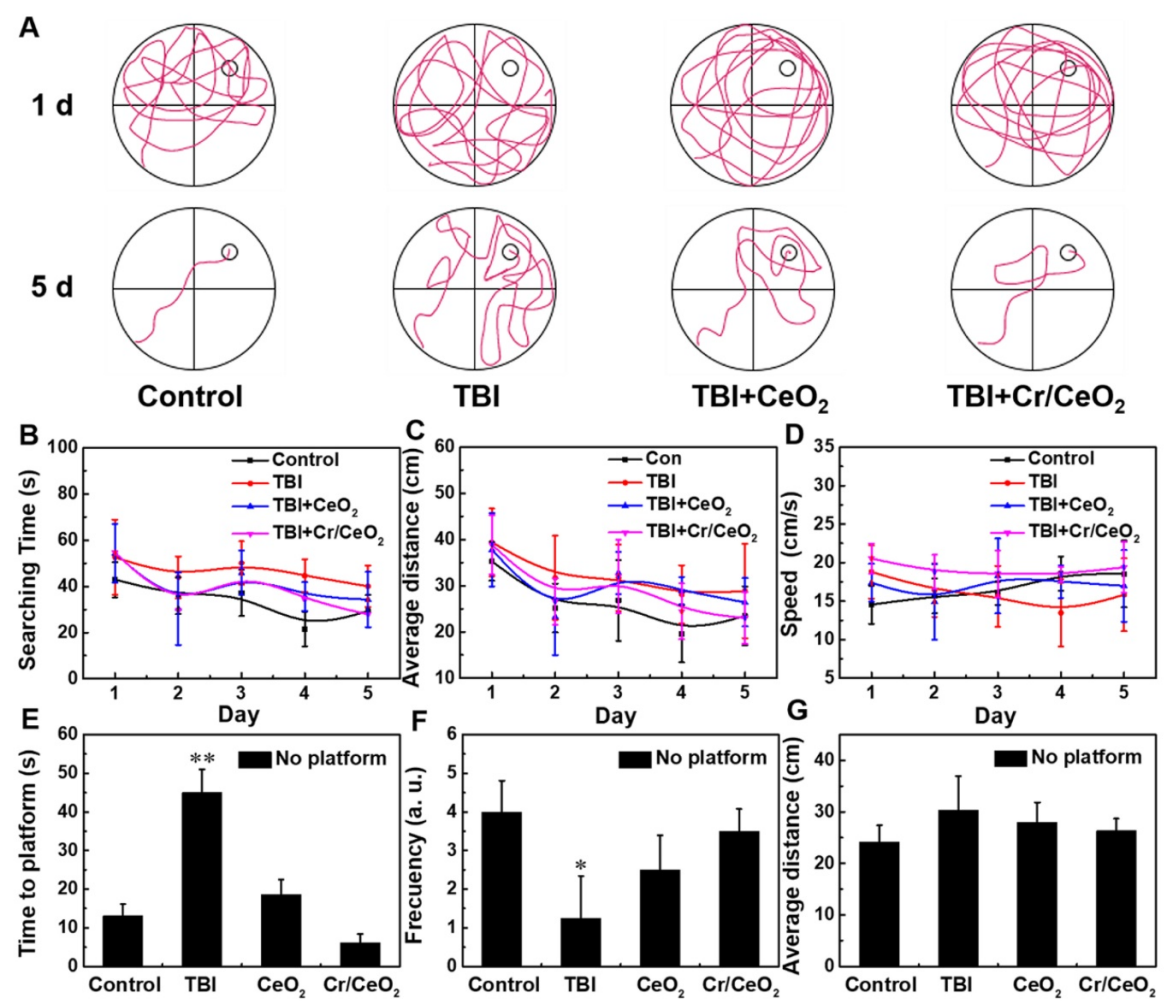
**Behavioral tests after TBI. A)** Path of the mice search platform in training on the first and fifth days. **B)** Time for searching the platform, **C)** average distance to platform, and **D)** swimming speed of mice during 5 days training. **E)** Time to find the initial hidden platform area for the first time, **F)** frequency of reaching the hidden platform area, and **G)** average distance from mice to the hidden platform area after the platform is removed on the fifth day test. All tests were performed on four groups: healthy control, TBI, TBI+CeO_2_ and TBI+ Cr/CeO_2_ nanozyme patch. Asterisks indicate significant differences (*P < 0.05, **P < 0.01, ***P < 0.001) as compared with the control group, analyzed by a Student's t test.

Finally, morris water maze test was performed to investigate spatial learning ability and memory ability of mice. **Figure 8A** shows that most of mice in all groups are unable to find the platform when they are trained on the first day. After 5 days of training, normal mice are able to quickly locate and land on the platform. The mice with Cr/CeO_2_ nanozyem patch treatment can successfully find the platform after a short period of time, while the mice in TBI group still do not quickly locate the exact position of platform. **Figure 8B**-**C** show that nanozyme patch-treated mice shorten the time for searching platform and stay closer to the platform during the 5-day training period, indicating that the mice after treatment have better learning ability compared with injured mice. Analyses of swimming speed of mice reveal that the mice with the treatment of Cr/CeO_2_ nanozyme patch are faster and closer to health mice (**Figure 8D**). After the platform is removed on the fifth day, the mice treated with nanozyme patch are able to quickly find and return to the location of the previous platform multiple times (**Figure 8E**-**F**). Therefore, behavioral testing demonstrates that the neuronal cognition of mice treated with nanozyme patch is improved, and their spatial learning and memory abilities are close to healthy mice. Finally, the blood chemistry and biochemical analysis were conducted on mice treated with or without nanozyme patch at 12 and 26 days post TBI, respectively, and compared with healthy mice. **Figure S12** and **S13** reveal that the mice treated with nanozyme patch have alleviated the abnormality of blood chemistry caused by TBI. Therefore, it can be seen that the administration mode of topical treatment has proven its feasibility in terms of safety and convenience, providing a promising noninvasive solution for future TBI treatment.

Nowadays, traditional treatments mainly performed by tail vein injection or intraperitoneal injection through the blood circulation 18, 66-69, accompanied by potential biosafety issues associated with the inherent toxicity of nanomaterials. It is essential to explore a new type of TBI treatment to reduce side effects. Herein, catalytic patch based on redox Cr/CeO_2_ nanozyme, a noninvasive topical treatment mode, was developed to fulfill this goal, significantly diminishing the accumulation of nanozyme in the brain without affecting its therapeutic effect. At the same time, the potential toxicity caused by doping of chromium, which is greatly reduced via this noninvasive treatment method. Moreover, the catalytic activity of previously reported ceria nanozyme is still not high enough, and the free radicals scavenging abilities are primarily directed to ROS 41. In this work, the obtained Cr/CeO_2_ nanozyme with multienzyme-like activities and possesses more removal affinity towards RNS, especially ONOO^-^ and DPPH, the secondary RNS with high reactivity and neurotoxicity 11. Therefore, it is desirable to promote the application of nanozyme patch into clinical therapeutic, including *in vivo* inflammation treatment, by increasing the biocompatibility of substrate materials. Furthermore, efforts will be focused on synthesis ultra-small size nanozyme at atomic precision levels to further elevate its GPx-like performance and the ability to selectively remove free radicals 70-75.

## Conclusion

In summary, the catalytic patch based on redox Cr/CeO_2_ nanozyme is developed for TBI therapy. The catalytic activity of nanozyme is optimized by modulating Cr^3+^ doping ratio. The Cr/CeO_2_ nanozyme possesses more effective RONS scavenging and multienzyme-like properties than undoped ceira, which is ascribed to the increase of Ce^3+^/Ce^4+^ ratio. *In vitro* experiments reveal the Cr/CeO_2_ nanozyme exhibits protection effects of cells against by LPS- and H_2_O_2_-induced damage. Meanwhile, *in vivo* experiments suggest that the nanozyme patch accelerates wound healing by improving SOD activity and reducing lipid peroxide. Tissue staining indicates that the treatment with nanozyme patch relieves excessive stress response of nerve cells in the brain. Furthermore, behavire test proves mice with nanozyme patch treatment exhibit strikingly neurocognitive recovery in spatial learning and memory abilities. Present work provides a noninvasive TBI therapeutic approach of nanozyme patch with minimized side effects.

## Supplementary Material

Supplementary figures.

## Figures and Tables

**Table 1:** Comparison of the Michaelis-Menten constant (*K_m_*)and maximum reaction rate (*V_max_*) between Cr/CeO_2_ nanozyme and HRP.

Nanozyme	Substance	*K_m_* [mM]	*V_max_* [µM/s]
Cr/**CeO_2_**	TMB	0.080±0.009	0.138±0.027
**Cr/CeO_2_**	H_2_O_2_	0.867±0.073	0.159±0.024
**HRP**	TMB	0.200±0.042	0.124±0.023
**HRP**	H_2_O_2_	0.665±0.063	0.112±0.016
