# Supplementary material for: Catalytic patch with redox Cr/CeO2 nanozyme of noninvasive intervention for brain trauma
Source: Theranostics. 2021 Jan 1;11(6):2806–21. doi: 10.7150/thno.51912 (PMC7806487; doi:10.7150/thno.51912)
Supplement: Supplementary file 1 — Supplementary figures. [file thnov11p2806s1.pdf]

Supplementary Material

Catalytic patch with redox Cr/CeO_2 nanozyme of noninvasive intervention for brain trauma

*Shaofang Zhang,[†, 1] Ying Liu,[†, 1] Si Sun,[†, 1] Junying Wang,[1] Qifeng Li,[4] Ruijuan Yan,[1] Yalong Gao,[4] Haile Liu,[1] Shuangjie Liu,[2] Wenting Hao,[2] Haitao Dai,[1] Changlong Liu,[1] Yuanming Sun,[3] Wei Long,[3] * Xiaoyu Mu,[2] * and Xiao-Dong Zhang,[1, 2] **

[1] Tianjin Key Laboratory of Low Dimensional Materials Physics and Preparing Technology, Institute of Advanced Materials Physics, School of Sciences, Tianjin University, Tianjin, 300350, China

[2] Academy of Medical Engineering and Translational Medicine, Medical College, Tianjin University, Tianjin, 300072, China

[3] Tianjin Key Laboratory of Molecular Nuclear Medicine, Institute of Radiation Medicine, Chinese Academy of Medical Sciences and Peking Union Medical College, Tianjin, 300192, China

[4] Department of Neurosurgery and Key Laboratory of Post-trauma Neuro-repair and Regeneration in Central Nervous System, Tianjin Medical University General Hospital, Tianjin 300052, China.

[†] These authors have contributed equally.

* Corresponding Author: Xiao-Dong Zhang (E-mail: **xiaodongzhang@tju.edu.cn**), Xiaoyu Mu (E-mail: **muxiaoyu@tju.edu.cn**) and Wei Long (E-mail: **longway@irm-cams.ac.cn**)

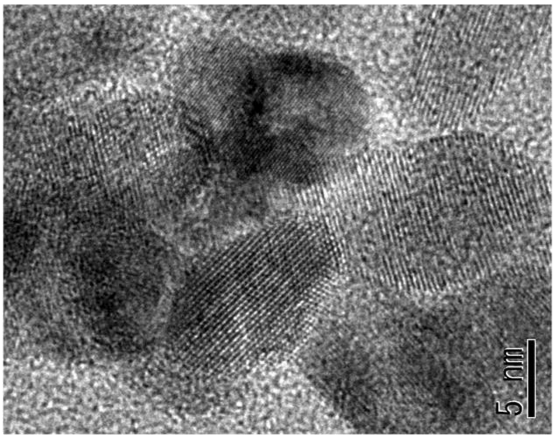

Figure S1. TEM image of Cr/CeO_2 nanozyme.

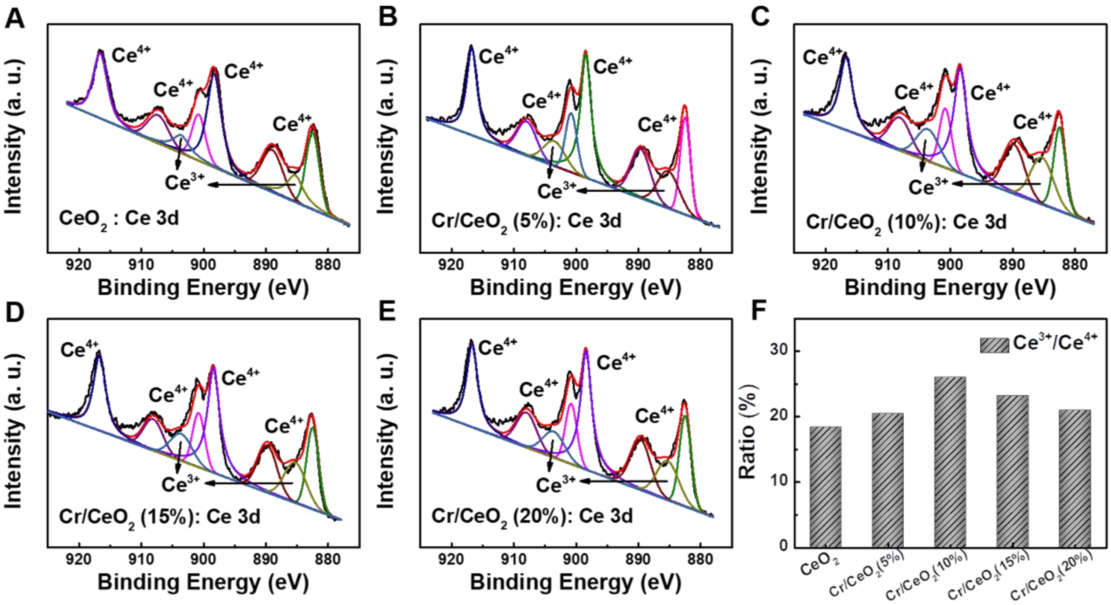

Figure S2. XPS spectra of **A)** CeO_2 and **B-E)** Cr/CeO_2 nanozymes with different doping concentration for Ce 3d. **F)** Ratio of Ce^{3+} /Ce^{4+} in CeO_2 and Cr/CeO_2 nanozymes with different doping concentration.

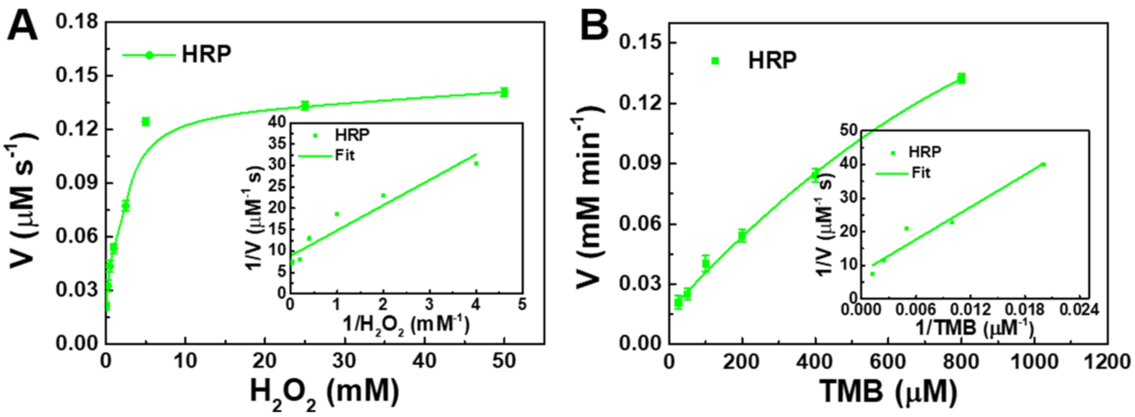

Figure S3. Steady-state Kinetic Assay of HRP. The velocity (v) of the reaction was measured using 0.02 ng/mL HRP. A) The concentration of TMB was 0.8 mM and varied concentration of H_2O_2. **B)** The concentration of H_2O_2 was 50 mM and varied concentration of TMB. Inset: Double-reciprocal plots of activity of HRP at a fixed concentration of one substrate versus varying concentration of the second substrate.

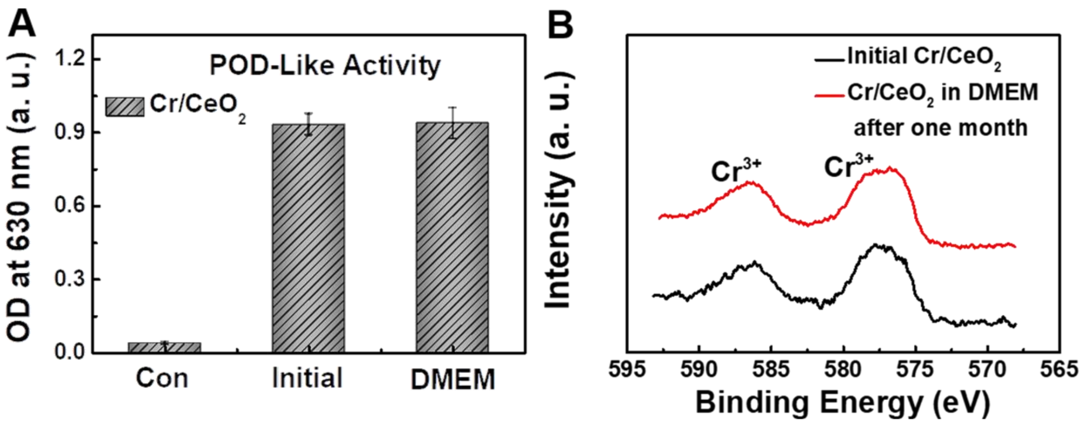

Figure S4. The stability of Cr/CeO_2 nanozyme in performance and structure. A) The POD-like activity of initial Cr/CeO_2 nanozyme or Cr/CeO_2 nanozyme in DMEM after one month. **B)** XPS spectra of initial Cr/CeO_2 nanozyme or Cr/CeO_2 nanozyme in DMEM after one month on Cr 2p.

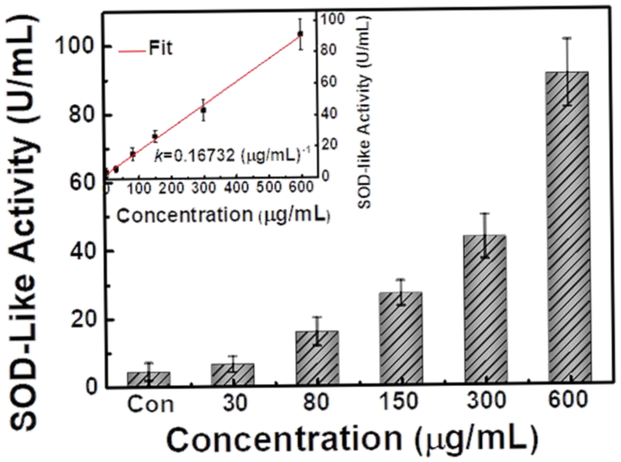

Figure S5. Concentration-dependent SOD-like activity of Cr/CeO_2 nanozyme in NBT assay.

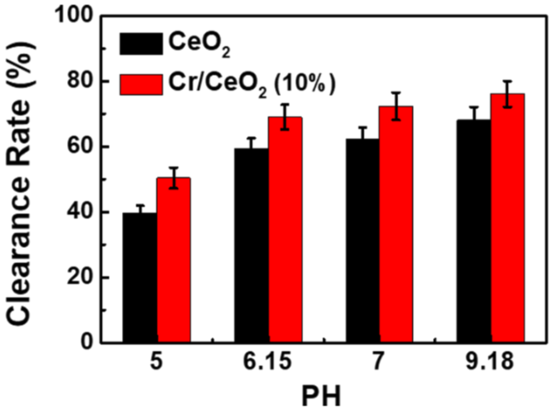

Figure S6. The H_2O_2 clearance rate of CeO_2 and Cr/CeO_2 nanozymes corresponds to the environment with pH values of 5, 6.15, 7 and 9.18, respectively.

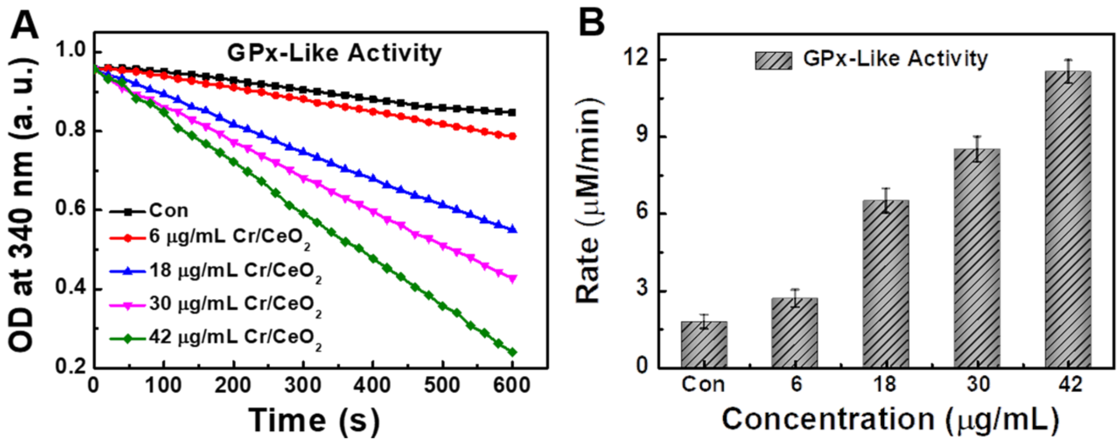

Figure S7. A) GPx-like activity of Cr/CeO_2 nanozyme at different concentration by GPx Assay Kit. **B)** Corresponding reaction rates of Cr/CeO_2 nanozyme at different concentration were calculated during GPx-like activity assay.

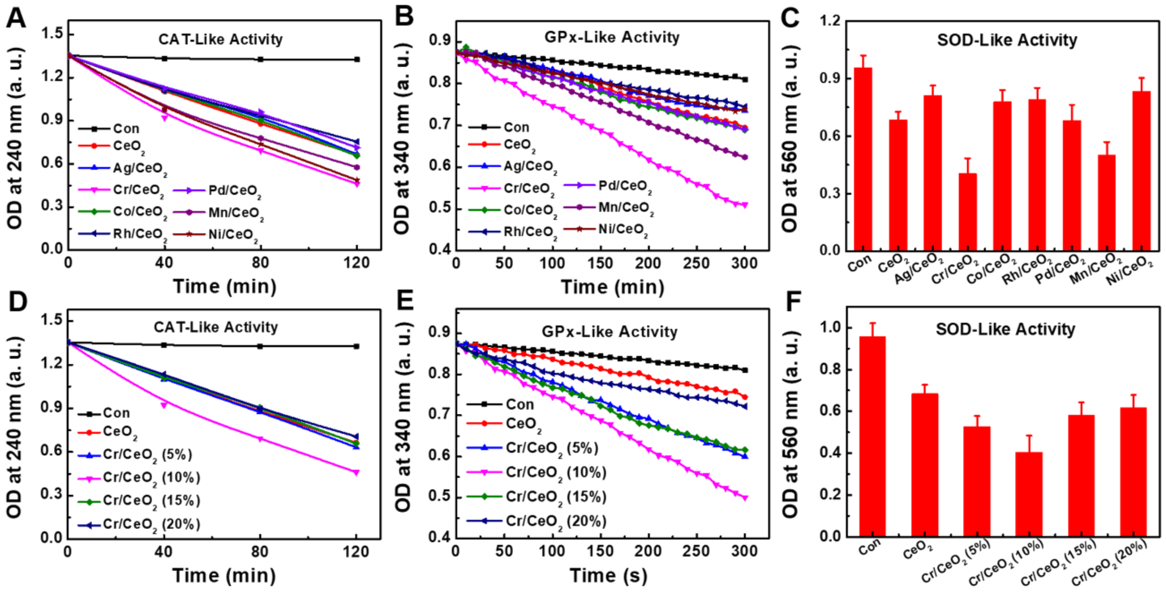

Figure S8. A) CAT-like, **B)** GPx-like and **C)** SOD-like activities of CeO_2 nanozyme doping with different metal elements. **D)** CAT-like, **E)** GPx-like and **F)** SOD-like activities of Cr/CeO_2 nanozyme with different doping concentration.

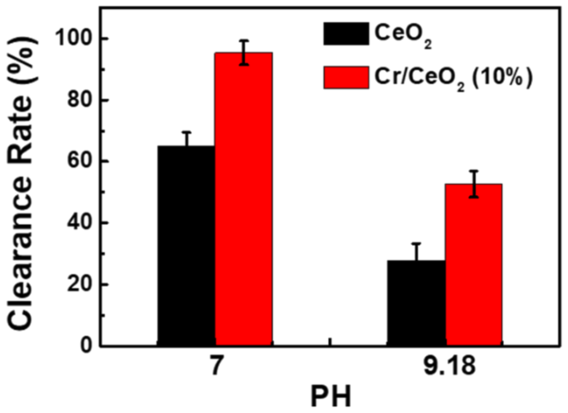

Figure S9. The $ONOO^-$ scavenging activity of CeO_2 and Cr/CeO_2 nanozymes corresponds to the environment with pH values of 7 and 9, respectively.

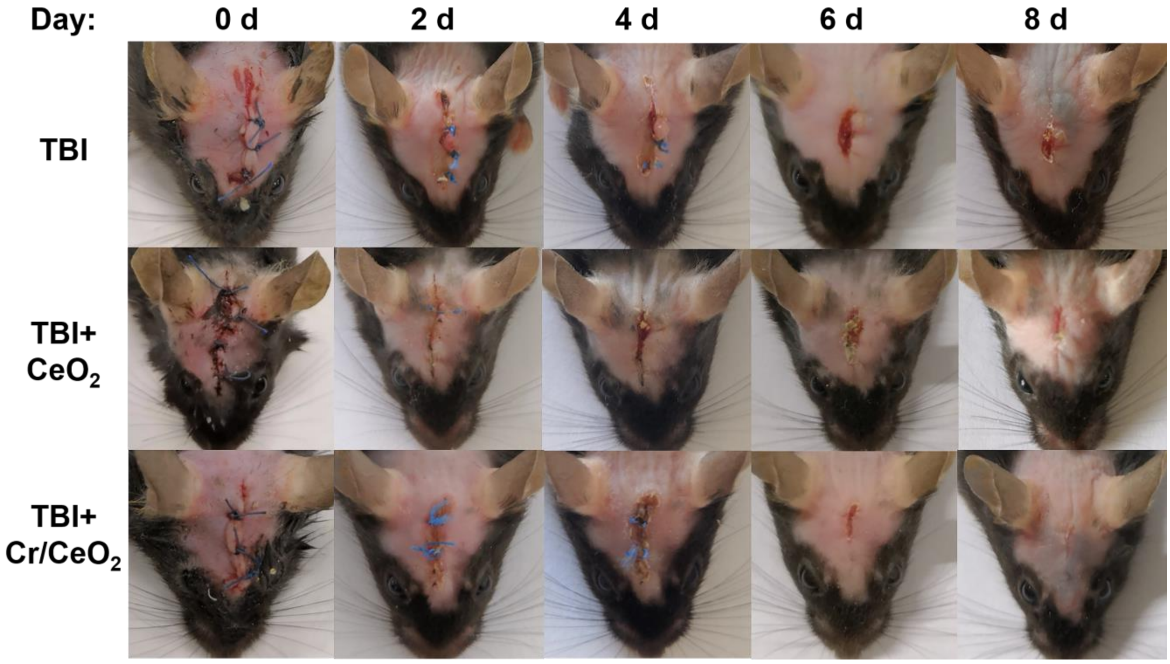

Figure S10. Photograph of mice wounds in TBI, TBI+ Cr/CeO_2 and TBI+Cr/CeO_2 groups over time.

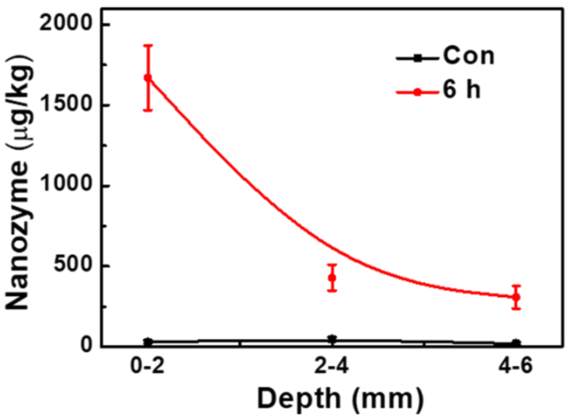

Figure S11. The concentration of Cr/CeO_2 nanozyme spread into the injured brain at different depths within 6 hours after patch-treated.

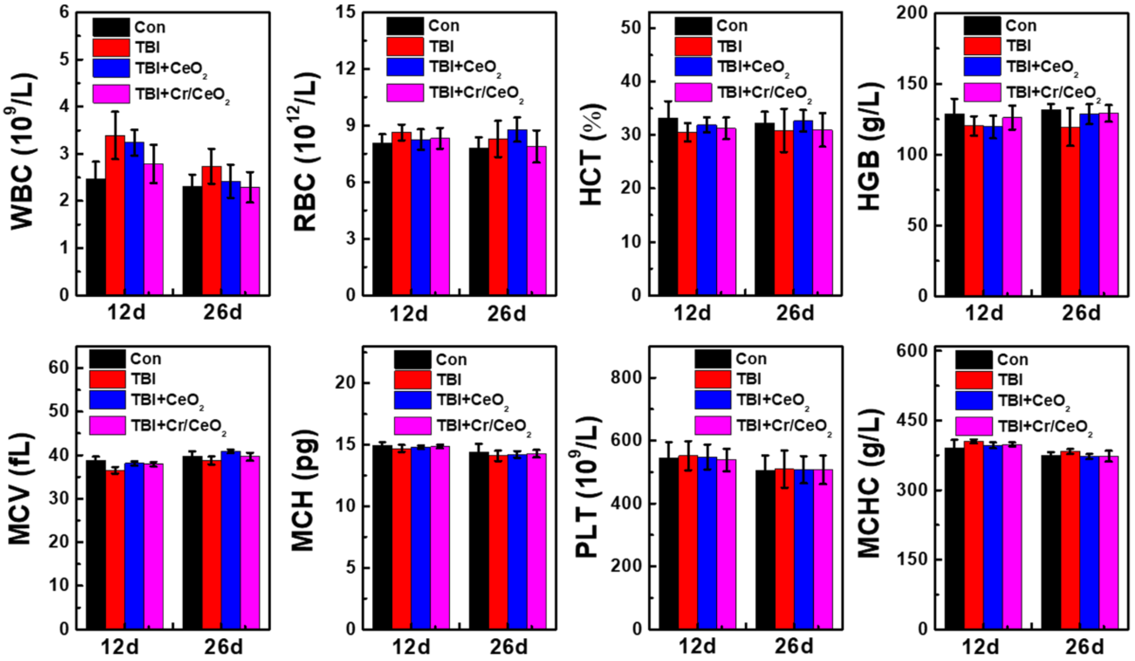

Figure S12. Hematologic data of mice in TBI, TBI+CeO_2 and TBI+ Cr/CeO_2 groups at 12 and 26 days after brain injure.

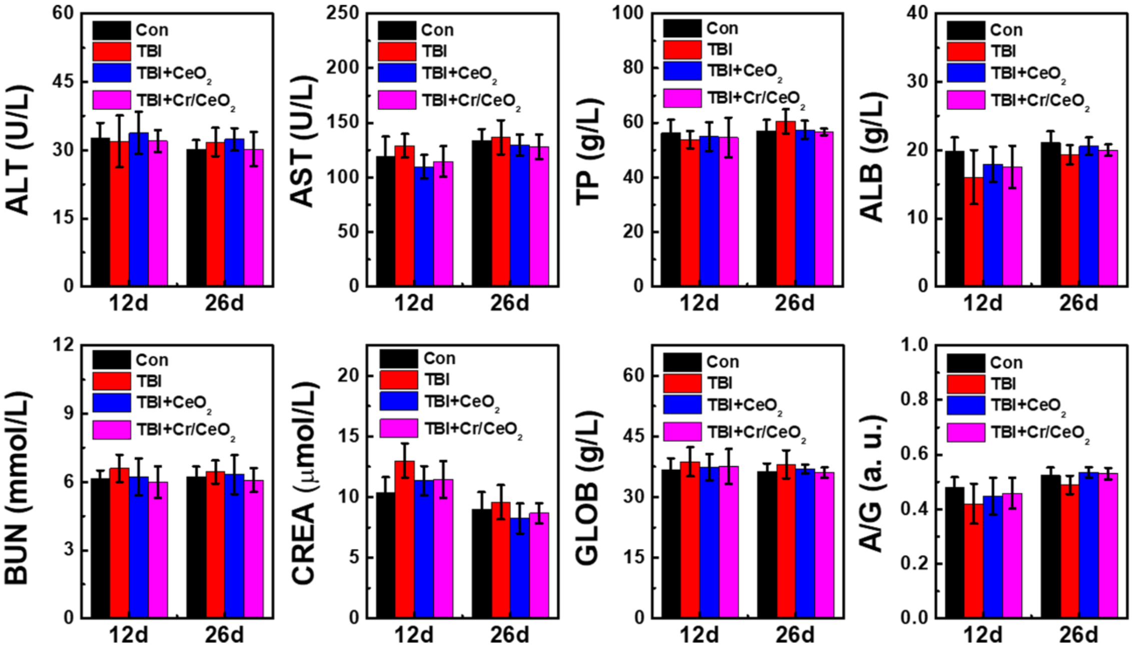

Figure S13. Blood biochemical data of mice in TBI, TBI+CeO_2 and TBI+Cr/CeO_2 groups at 12 and 26 days after brain injure.
